# Genetic variation in the Middle East—an opportunity to advance the human genetics field

**DOI:** 10.1186/s13073-020-00821-7

**Published:** 2020-12-28

**Authors:** Ahmad N. Abou Tayoun, Heidi L. Rehm

**Affiliations:** 1 Al Jalila Genomics Center, Al Jalila Children’s Hospital, Dubai, United Arab Emirates; 2 College of Medicine, Mohammed Bin Rashid University of Medicine and Health Sciences, Dubai, United Arab Emirates; 3 Medical & Population Genetics Program and Genomics Platform, Broad Institute of MIT and Harvard, Cambridge, MA 02142 USA; 4 Department of Pathology, Harvard Medical School, Boston, MA 02115 USA; 5 Center for Genomic Medicine, Massachusetts General Hospital, Boston, MA 02114 USA

## Abstract

We highlight the current lack of representation of the Middle East from large genomic studies and emphasize the expected high impact of cataloging its variation. We discuss the limiting factors and possible solutions to generating and accessing research and clinical sequencing data from this part of the world.

## Genomic diversity in large-scale sequencing efforts and persistent bias

In a series of landmark articles, the largest sequenced human cohort representing a diverse group of individuals from across the world has been characterized. The Genome Aggregation Database (gnomAD) represents aggregated and uniformly processed whole genome (*n* = 15,708) and exome (*n* = 125,748) sequencing data to catalog genetic variation across coding and noncoding regions of the human genome [1, 2]. The dataset, which now includes structural variants (SVs), as well as 241 million small variants, empowers researchers to estimate gene tolerance to variation in an unprecedented way, and aids in the clinical interpretation of genome variation [1, 2].

**Fig. 1:**
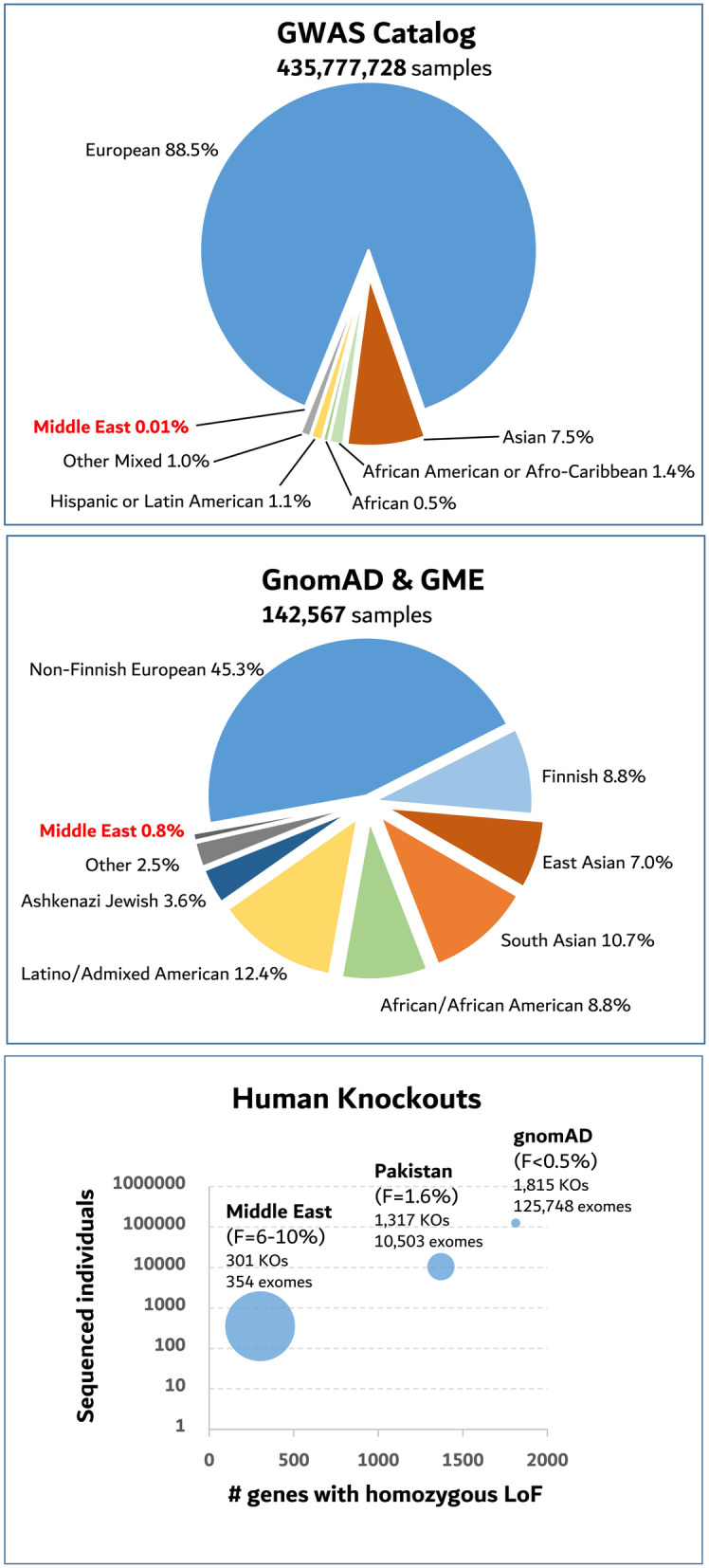
Genomic data representation. *Top*, Distribution of sample ancestries in the GWAS Catalog as of June 14, 2020 [5]. *Middle*, Distribution of sample ancestries in the Genome Aggregation Database (gnomAD v2.1) [1] and the Greater Middle East (GME) variome [6] studies. *Bottom*, Human knockouts (KOs) or the number of genes with homozygous loss of function (LoF) variant(s) in exomes from individuals of the Greater Middle East [6], Pakistan [7], and gnomAD v2.1 [1]. Circle size correlates with a fraction of the number of KOs in a given sample size. *F* = coefficient of inbreeding

One of the main strengths of the gnomAD database, and its predecessor, the Exome Aggregation Consortium (ExAC) database [3], lies in capturing sequencing data representing diverse European and non-European ancestries at a larger scale compared to previous sequencing studies [4]. Around 43% of individuals in the gnomAD database are non-European Asians (10.8% South Asians and 7% East Asians), Latino (12.5%), Ashkenazi Jewish (3.7%), and Africans or African Americans (8.8%) (Fig. 1). This represents a major shift from the existing significant bias in most large-scale genomic studies, where the majority of individuals have been mostly of European origin, raising legitimate concerns that precision or genomic medicine will be a privilege for the “few” represented in those studies. As of June 14, 2020, 88.5% of genome-wide association studies (GWAS)—summarized in the GWAS Catalog (www.ebi.ac.uk/gwas), produced by the US National Human Genome Research Institute and the European Bioinformatics Institute, and recently monitored through the GWAS Diversity Monitor (www.gwasdiversitymonitor.com) [5]—were Europeans while only 7.5% were Asians and 4% Africans, Latin, and among few others (Fig. 1). Thus, gnomAD is an important step towards, a very much needed, broader genetic representation.

However, despite its size, the gnomAD dataset captures only 3.7 and 11.5% of all possible nonsense and synonymous variants, respectively, across all mutational contexts [1], thus falling short on identifying the full mutational spectrum of the coding regions. Increasing cohort size as well as further diversifying the genomic data to include individuals of unrepresented ancestries can help in this regard. While there has been progress in the growth of data across a number of ancestries, gnomAD authors correctly point out, this cohort has an almost complete absence of representation from certain groups, mainly Oceania, Southeast Asia, much of Africa, and the Middle East [1]. Among these, Arabs of the Middle East, geographically spanning North Africa, the Arabian Peninsula, and the Syrian desert, have a rich history of migration and admixture with Africans, Europeans, Persians, Turks, and Southeast Asians. Besides Arabs, this region is also home for other minorities including Jews, Armenians, and Kurds.

## Missed opportunities in Arab genomes

In addition to genetic diversity, the Arab family is characterized by its large size, high maternity and paternity ages at conception and significant endogamy with consanguinity rates between 25 and 60% which are 100-fold higher than the < 0.2% consanguinity rate in Western countries [6]. Therefore, it is expected that Arab genomes have a high burden of regions of homozygosity (ROH) leading to a higher incidence of Mendelian recessive disorders [6].

From a population genomics standpoint, the extended ROH regions can be enriched for two-hit gene knockouts (homozygous loss of function, LoF, variants), in apparently “healthy” individuals, providing opportunities to understand the biological roles of several genes which cannot be encountered in outbred populations, like most data in gnomAD. Of 125,748 exomes in gnomAD v2.1, authors identified only 1815 genes with at least one homozygous LoF event [1], with the projection that a 1000-fold larger sample size would be needed to ascertain homozygous LoF of most genes. Furthermore, knockout for around 25% of the genes would not be encountered in this outbred population even if all humans on earth were sequenced [2]. While such knockouts might be lethal, larger outbred sample sizes are still needed to establish this. On the other hand, ascertainment of complete gene knockouts will be significantly enhanced by sequencing consanguineous cohorts [2], where the burden of homozygous gene LoF per individual correlates with the coefficient of inbreeding (F) as shown in one study, which identified 1317 bi-allelic gene knockouts in an inbred Pakistani cohort consisting of only 10,503 individuals with a median *F* value of 1.6% (versus 0.4% in Europeans and African Americans). This study anticipated the identification of 8754 gene knockouts if 200,000 individuals were sequenced from this inbred population [7]. The number of homozygous gene LoF is expected to be even higher in populations with diverse genetic composition and extensive inbreeding, like in the Middle East. In fact, whole exome sequencing data from 77 individuals born to first-cousin marriages in Saudi Arabia revealed on average 22.8 bi-allelic LoF variants per individual compared to 14.4, 15.9, and 14.3 homozygous LoF variants in individuals with European, Chinese or Japanese, and Nigerian ancestries, respectively [8]. Additionally, a slightly larger, and more representative, number of exomes (*n* = 354) from verified healthy adults in the Greater Middle East (GME), with *F* values between 6 and 10%, contained rare homozygous LoF variants in 301 genes, most of which (*n* = 207) did not overlap with complete knockouts from 60,706 individuals in ExAC [3, 6] (Fig. 1).

It is thus unquestionable that expanding sequencing studies within the Middle East will be a unique asset for the human genetics field due to their enrichment for autozygosity which can inform recessive gene-disease associations [9] and variant interpretation [10]. Unfortunately, however, this region remains poorly represented in large genomic studies. To date, only 0.01% of total participants in GWAS studies were from the Middle East, while almost none were included in gnomAD v2.1. Even if sequencing data from both the GME study mentioned above and the gnomAD v2.1 dataset were combined, this highly diverse region will represent less than 1% of all publicly accessible sequencing datasets (Fig. 1). This underrepresentation might be attributed to several factors which have to be addressed if we are to fully harvest genomic variation in the Middle East, which will in turn further our understanding of genetic diseases.

## Roadblocks in genomic sequencing in the Middle East

A major factor limiting genomic studies is the lack of comprehensive educational and awareness genetics programs in most Middle Eastern countries. Such programs are needed at every level to address cultural, legal, public health, and training issues associated with genomic investigations. There are societal stigmas attached to genetic diseases, discouraging families from pursuing any research or clinical genetic investigations. Trained genetic counselors, who are familiar with local traditions, are currently lacking but desperately needed to mitigate the stigmas surrounding genetics. Healthcare professionals are urged to educate decision-makers about the long-term societal and economic burdens of genetic diseases, so as to invest in establishing relevant genomic research and training programs in those countries. Outcomes of such programs include training local professionals in genomics, and building the genetic evidence to guide public health efforts, including national genetic screening programs, to be implemented in healthcare systems. Those efforts will eventually promote the implementation of genomic medicine, will better educate the public about genetics, and will lead to the proliferation of sequencing datasets to support research in this field.

Another limiting factor is obviously the lack of resources to enroll genomics into long-term research and healthcare agendas. Several countries in the Middle East are devastated by political tensions, economic crises, and regional or local conflicts. Under such circumstances, resources are inevitably allocated to other basic priorities leaving behind no room for investments in genomics, and hardly any relevant human resources. For some of those countries, a short-term solution might include collaborative projects with external investigators and institutions where funding agencies would encourage ascertainment of participants from the Middle East for genomic sequencing studies.

Given the above roadblocks, genomic studies are limited to very few laboratories—in a few countries—where sequencing data exist in silos, and are not broadly shared, most likely due to existing stigmas and regulatory restrictions around genetic data, which end up hampering the research consent process. One goal of the educational programs discussed above would be to encourage the public, researchers, and legislators to broadly share genetic data nationally and internationally with the common ultimate goal of advancing the human genetics field, and expanding our knowledge of genetic diseases. Failing to do so risks the national interests of any country by depriving its residents of the long term advances in genomics.

Finally, outside the Middle East, it is important to distinguish “Arabs” from “White” ancestries, as is currently considered by the US Government Consensus. This might lead to better representation of “Arabs,” given the US efforts to enhance the representation of “minorities” in genomic databases.

## Conclusions

Broadly unlocking the Middle Eastern Arab—and other minority—genomes promises to advance human genetics research, and bolster precise genomic diagnostics and therapeutics. It is encouraging that national genome projects started developing in the Gulf region (Kuwait, Qatar, Saudi Arabia) which will hopefully expand to other countries and lead to sharing broader and more representative genetic data from the Middle East. We all share most of the human genome sequence, and we can only understand it better if we collectively share our diverse genetic variation.
